# Using a human-centred design approach to develop a comprehensive newborn monitoring chart for inpatient care in Kenya

**DOI:** 10.1186/s12913-021-07030-x

**Published:** 2021-09-24

**Authors:** Naomi Muinga, Chris Paton, Edith Gicheha, Sylvia Omoke, Ibukun-Oluwa Omolade Abejirinde, Lenka Benova, Mike English, Marjolein Zweekhorst

**Affiliations:** 1 Athena Institute, VU University Amsterdam, Amsterdam, Netherlands; 2 KEMRI/Wellcome Trust Research Programme, Nairobi, Kenya; 3 Sexual and Reproductive Health Group, Department of Public Health, Institute of Tropical Medicine, Antwerp, Belgium; 4 Centre for Tropical medicine and Global Health, Nuffield Department of Medicine, University of Oxford, Oxford, GB England; 5 Department of Information Science, University of Otago, Dunedin, New Zealand; 6 Rice University, Houston, USA; 7 Dalla Lana School of Public Health, University of Toronto, Toronto, Canada

**Keywords:** Human-centred design, Nursing care, Newborn, Inpatient, Documentation

## Abstract

**Introduction:**

Job aids such as observation charts are commonly used to record inpatient nursing observations. For sick newborns, it is important to provide critical information, intervene, and tailor treatment to improve health outcomes, as countries work towards reducing neonatal mortality. However, inpatient vital sign readings are often poorly documented and little attention has been paid to the process of chart design as a method of improving care quality. Poorly designed charts do not meet user needs leading to increased mental effort, duplication, suboptimal documentation and fragmentation. We provide a detailed account of a process of designing a monitoring chart.

**Methods:**

We used a Human-Centred Design (HCD) approach to co-design a newborn monitoring chart between March and May 2019 in three workshops attended by 16–21 participants each (nurses and doctors) drawn from 14 hospitals in Kenya. We used personas, user story mapping during the workshops and observed chart completion to identify challenges with current charts and design requirements. Two new charts were piloted in four hospitals between June 2019 and February 2020 and revised in a cyclical manner.

**Results:**

Challenges were identified regarding the chart design and supply, and how staff used existing charts. Challenges to use included limited staffing, a knowledge deficit among junior staff, poor interprofessional communication, and lack of appropriate and working equipment. We identified a strong preference from participants for one chart to capture vital signs, assessment of the baby, and feed and fluid prescription and monitoring; data that were previously captured on several charts.

**Discussion:**

Adopting a Human-Centred Design approach, we designed a new comprehensive newborn monitoring chart that is unlike observation charts in the literature that only focus on vital signs. While the new chart does not address all needs, we believe that once implemented, it can help build a clearer picture of the care given to newborns.

**Conclusion:**

The chart was co-designed and piloted with the user and context in mind resulting in a unique monitoring chart that can be adopted in similar settings.

**Supplementary Information:**

The online version contains supplementary material available at 10.1186/s12913-021-07030-x.

## Background

The global under 5 mortality rate has declined to 38 per 1000 live births in 2019 from 76 in 2000, with nearly half of these deaths in newborns [1]. Many countries are working toward the Sustainable Development Goal (SDG) 3.2 target of reducing neonatal mortality to 12 per 1000 live births [2]. In sub-Saharan Africa, the newborn mortality rate remains high at 27 deaths per 1000 live births in 2019 [1]. Most of these deaths are caused by preventable and treatable illnesses, such as preterm birth complications, birth asphyxia, pneumonia, congenital anomalies, diarrhoea, and malaria, emphasizing the need to strengthen health systems [3]. Furthermore, the percentage of births occurring in health facilities, including hospitals, has been increasing rapidly with the move toward Universal Health Coverage (UHC) [4]. Hospitals also now provide intensive neonatal care and need to be well equipped to provide care for small and sick newborns, whether born on-site or referred [5].

### Inpatient newborn nursing care

As part of efforts to reduce neonatal mortality, high quality, round-the-clock care for newborns who can spend many days in the neonatal unit is required [6–8]. While newborn care is often planned by a multidisciplinary team, nurses are the primary caregivers. They have the greatest patient contact, placing them at the heart of information generation and archiving, which is necessary for continuity of care and team communication [9]. For sick newborns, documenting vital sign observations, feed and fluid prescription and monitoring, and weight gain are particularly important to provide critical information for communicating, intervening, tailoring treatment, and improving mortality and morbidity outcomes [8, 10].

### Documentation of newborn care

To facilitate documentation of nursing care, nursing observations may be recorded using job aids, which are tools to help structure, standardize, and facilitate work processes. The function of job aids is to extend cognitive capacity by removing extraneous details in the care process, resulting in simplified procedures or tasks. They minimize the need for reliance on memory to document details of nursing actions and plans [11]. Examples of job aids include rounding checklists used in intensive care units [12], structured forms for recording patient observations (such as the pediatric admission record, − PAR) [13], patient safety checklists [14, 15], and forms guiding patient handover between staff shifts [16].

### Design of charts for documenting nursing care

Poorly designed or inadequate job aids may have the opposite effect, increasing mental effort and failing to meet the needs of the users, resulting in problems such as duplication of efforts [17, 18], fragmentation [8, 19], non-use or suboptimal use and ‘improvisation’ of documentation, such as the use of ‘scraps’ (of paper) for handover [20]. Nurses in general, and particularly those in neonatal care units, spend a large proportion of their time documenting care [21, 22]. Considering that health facilities, especially in low- and middle-income countries, are already short-staffed, the burden of documenting might be eased by well-designed job aids or charts. For observation charts to facilitate the provision of quality care and highlight core clinical care activities, they should be designed to meet the needs of their different users, whether to document providers’ actions or to gain information on what has (or has not) been done for the patient. Additionally, such charts should: i) consider providers’ working context, ii) promote professional standards for quality care, and, iii) minimize the burden of documentation activities.

Research from both high- and low- and middle-income settings has shown that vital signs are often poorly documented in observation charts. Detection of deteriorating physiological signs in hospitalized patients is suboptimal either because of poor chart design [23] or poor understanding of why vital signs are measured and documented [24, 25]. However, little attention has been paid to the design of inpatient monitoring charts [26] and their contribution to the completeness of documentation and quality of care.

### Documentation of nursing care in Kenya

In Kenya, nurse administrators reported that documentation of nursing care remains a challenge [17]. Specifically for newborns - a vulnerable group of patients - poor documentation of care also limits the use of routine data for quality improvement [27], making it a priority area for improvement. Recently, the need was identified in hospitals in Kenya for better tools to document newborn nursing observations that would facilitate rapid, accurate, and informative communication between nurses and other professionals as a key part of improving the quality of care [8].

### Objective

In this article, we describe the progress and outcomes of a process aimed at improving the documentation of newborn care within a network of hospitals in Kenya, involving nurses and other professionals and applying an adapted Human-Centred Design (HCD) approach to the design process. This article also fills a gap identified in a recent scoping review that found limited detailed literature on the application of HCD projects [28].

## Human-Centred design approach

Human-Centred Design (HCD), “is an approach that puts human needs, capabilities, and behaviour first, then designs to accommodate those needs, capabilities, and ways of behaving.” ([29] Pg 14).. The approach seeks to engage stakeholders, understand the context, explore solutions, test them, and implement them in a cyclic process. It is also founded on the premise that those who face a problem every day are likely to hold the key to its solution [30].

Historically, the Human-Centred approach, which encompasses various design approaches such as design thinking and user experience design, has been applied by businesses to design products, restructure work environments and build a better understanding of clients [31, 32]. The approach has most commonly been used to create interactive user interfaces in software development, where usability is a critical factor of design [33]. More recently, this approach has been applied in the design of sustainable malaria interventions [32, 34] and social innovations [28, 31]. Paper-based monitoring charts are interfaces for health workers and may form templates for electronic medical records, lending themselves to applying similar design approaches.

### The adapted human-Centred design

**Fig. 1:**
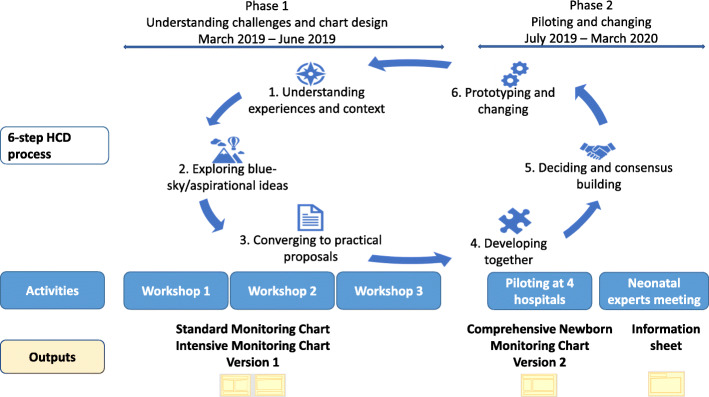
Chart design process

The Human-Centred Design applied in this research is adapted from the design processes of Boyd et al. [35] (six-stage) and Bowen et al. [36] (five-stage). We used a 6-step process in two phases: design and piloting (Fig. 1). Phase one began by understanding the monitoring experience and exploring ideas on how to address the challenges identified. Next, we focused on practical proposals given the context and developed these ideas together with the stakeholders, nurses, and doctors, resulting in version 1 charts. In phase two, we included the prototyping and changing phases of Boyd’s process; we piloted and adapted the charts based on feedback, leading to version 2. While the process is described sequentially, it was cyclical: participants reflected upon earlier suggestions and revised them with subsequent in-workshop chart tests and feedback from piloting and as contextual issues became clearer. Figure 1 illustrates the design process with the activities and outputs in each phase. We did not develop the blue-sky concepts in Bowen’s process as our time with participants was limited; it was not a problem because the aim was to develop a product that improves on existing charts.

Under the HCD approach, various design methods can be applied to achieve the overall goal of placing the user at the core of the design. In our design workshops, we adapted the scrum methodology based on Agile software development principles, which are usually used to develop software in iterative and incremental cycles to quickly produce a minimum viable product (MVP) that can be tested (steps 3–6) [37]. Additionally, we used Lean User Experience (LeanUX) concepts, focusing on understanding the user, the context, and how the product is used through personas and user story mapping (step 1) as well as designing with the user [38]. Participatory design activities and the scrum methodology allowed us to develop and test the product as we went along, rather than accept a final product early on. We adapted these concepts to suit our setting, such as by pre-identifying personas likely to use the chart based on previous experience in hospitals, before the short workshops. We also used an adapted form of journey maps [39] or user story mapping [40] together with a guide to understanding the monitoring task and its challenges.

## Methods

### Setting

This study leverages an established Clinical Information Network for Newborns (CIN-N) comprising 20 county referral hospitals located in Nairobi, the Central, Eastern, and Western parts of Kenya. CIN-N is a partnership between researchers, the Kenya Ministry of Health, and paediatricians, providing a research database of patient-level data from all neonatal admissions upon discharge [41–43]. Collected data include clinical presentation, immediate treatment, and discharge information and are intended to improve the use of information in policy and practice. This study complements the aims of the CIN-N by developing observation charts for documenting vital signs, tracking feed, and fluid prescription and administration, contributing to better tracking and monitoring of inpatient neonatal care within and across hospitals. The hospitals in the network do not have neonatal intensive care units; their annual range of inpatient volume was 322 to 3788 in 2019. The newborn unit staff comprises 1–7 paediatricians or a neonatologist per hospital and 7–21 nurses (administrative and clinical). Previous work has reported a median ratio of 1 nurse to 19 babies [44]. Other staff included student nurses, medical officers (clinically qualified but no specialization), medical officer interns, clinical officers (non-physician clinicians), clinical officer interns, and nutritionists. All hospitals received students from nearby training facilities.

We conducted three workshops with 16–21 participants each between March and May 2019 in phase one (Table 1). The participants, senior nurses and paediatricians drawn from 14 CIN-N hospitals, were purposely selected as managers of neonatal wards who could decide what chart designs would suit their context. We also received feedback from other health professionals in the four piloting hospitals that were on duty on the day of the pilot visit. Detailed field notes and meeting summaries were maintained during the workshops and piloting phase. These notes were used together with personas, and journey maps to inform chart design at all stages. Workshops and feedback sessions were all conducted in English.

**Table 1:** Summary of workshops in phase one

Workshop	Workshop tasks	Participants(no)	Dates and duration	Outputs	Number of CIN-N hospitals represented
**Workshop 1** Focus on nursing tasks: feed, fluid, and vital signs monitoring	• Identifying challenges with current monitoring charts • Describing personas (nurse and clinical officer) • Exploring design solutions to a new chart	Senior nurses (20)	March 2019 Duration: 3 h Setting: part of a CIN-N meeting with related agenda	• Problem list • 2 Personas • Design ideas	13
**Workshop 2** Focus on feed and fluid prescribing task	• Describing personas (medical officer intern) • Describing feed/fluid prescription task	Paediatricians (16) Senior nurses (4) Medical Officer (1)	April 2019 Duration: 3 h Setting: CIN-N dedicated session at Kenya Paediatrics Association conference	• Problem list • 1 Persona • Design ideas	14
**Workshop 3** Focus on chart content	• Describing the process of monitoring feeds and fluids and identifying challenges • Designing 1st prototype • Testing prototype using scenarios	Senior Nurses (16)	May 2019 Duration: 1.5 days Setting: dedicated chart design workshop	• Process flow diagram • Monitoring charts – version 1	12

### Phase one – understanding the user needs, goals and context and tool design

Before the workshops, the study team obtained uncompleted and completed de-identified monitoring charts from a selection of newborn units within the CIN-N to identify existing charts used for monitoring, to understand how they were filled, and the challenges encountered in using them.

#### Understanding the user needs, goals, and context

##### Personas

Personas are fictional characters based on research or real-world experiences that represent user types that might interact with a product [45]. Personas guide designers to articulate user demographics, needs, and behaviours; they include a sketch that personalizes the description. In workshops 1 and 2, we used personas to understand user needs, experiences, behaviours, and goals. In groups, workshop participants identified documentation challenges and described three pre-identified personas: a nurse providing round-the-clock monitoring care, a clinical officer, and a medical officer intern, who were staff most likely to admit babies [46] and therefore likely to write a feed and fluid prescription (Table 1). Each group discussion was facilitated by a member of the study team with experience working in newborn units as a paediatrician or nurse, and another with experience using HCD to design products. Facilitators played a facilitator-participant role even though their primary task was to help workshop participants articulate their ideas; we believe this contributed to open discussions during the workshop.

##### User story mapping

In workshops 2 and 3, participants described the monitoring process in scenarios (Table 2) using a guide developed by the study team to help them articulate how the personas would document care given to newborns by adapting user stories and journey maps. Working in three groups, workshop participants recorded what they required for the monitoring task and documentation (e.g. charts, reference materials, equipment), where they got it, where they recorded information, who it was aimed at, how it was used, and any other challenges they encountered. Two tasks served as examples: a) feed and fluid prescriptions written by the clinical team, and b) monitoring tasks performed and documented by nurses. The categorisation of babies reflects the level of dependency of neonatal inpatients that was specified as part of efforts to standardise neonatal care and determine the frequency of nursing tasks (8).

**Table 2:** Scenarios used in phase one

Scenario 1: Monitoring 1 (student/less experienced nurse)	Scenario 2: Monitoring 2 (student/less experienced nurse)	Scenario 3: Senior nurse review
Category A baby weighing 1.3 kg who is on intravenous fluids; the nurse is to check the baby and chart the fluid intake/output/vitals etc. at 3 pm	Category B baby weighing 1.7 kg who is on nasogastric tube feeding; the nurse is to check the baby and chart feeding/output/vitals etc. at 3 pm	A senior nurse who will do a handover to an expert colleague of a Category A baby including the progress in terms of input/output, vital signs monitoring, and other care

Three scenarios (Table 2) were designed to provide participants with situations they might typically encounter in the ward, including when the chart would be used and by whom: i) Category A baby – sick and requiring close monitoring, ii) Category B baby – more stable, requiring 3–6 hourly monitoring, and iii) a senior nurse who reviews the care provided to several babies.

#### Designing together

We explored design ideas in all workshops by asking participants to visualize an ideal chart (steps 2–3). In workshop 3, the participants (nurses) were provided with sample monitoring charts from hospitals within the network, private hospitals in Kenya, and newborn units outside Kenya (UK, Malawi, and India). Only nurses participated as the design workshop was combined with a communication training to use nurses’ time efficiently. In the three groups, participants focused on specific sections of the chart: 1) biodata and vital signs monitoring plus assessment, 2) feed monitoring, and 3) fluid monitoring. Facilitators helped participants identify and agree on which items were important to capture, and on appropriate field types for each item, for example, where Yes/No fields could be implemented, or a limited number of choices and which choices. Where there were well-known and accepted abbreviations, participants were encouraged to think about them critically and agree on their use, bearing in mind possible (mis)-interpretation. Each group then explained their design ideas to the others in a plenary session to reach a consensus on the fields captured and to identify any missing items.

The study team members then developed two monitoring charts: a) a standard monitoring chart for babies requiring 3–6 hourly checks and b) an intensive monitoring chart for babies requiring more frequent checks (not for use in the Neonatal Intensive Care Unit (NICU)), incorporating all workshop information. Finally, workshop participants used the three scenarios (Table 2) and the prototype monitoring chart to fill in a 48-h admission episode. This activity enabled them to identify where improvements were needed and to visualize a completed chart.

During the design sessions, participants were provided with the personas and reminded to keep in mind the user they were designing for and their context. Constantly referring to the personas and contextual realities helped the participants to reflect on whether suggestions were feasible as the charts were revised (steps 3–5).

### Phase two -piloting and changing

The two newly developed charts (standard monitoring chart and intensive monitoring chart – version 1) were piloted in four CIN-N hospitals (Three county and one tertiary hospital) between June 2019 and February 2020, which volunteered to pilot the chart after workshop 3. Additionally, one nurse implemented version 1 of the chart in her ward even before the piloting sessions began and reported that staff were happy to use it. The tertiary hospital had separately embarked on a project to update their existing charts and saw this as an opportunity to use a systematically developed chart. Using the version 1 chart as a starting point, we adapted the charts to the contextual realities of the pilot sites (e.g. a chart for the Neonatal Intensive Care Unit (NICU) in the tertiary hospital).

The four newborn units received 25 copies of each chart and nurses made additional copies to ensure all babies had a new chart to replace the usual charts. The senior nurses who had attended the workshops first explained to the staff how to use the charts, before distributing them. After 1–2 weeks of use, we conducted a total of eight site visits to pilot hospitals to receive feedback. Each lasted 45–90 min, attended by staff who had used the charts, including the senior nurse, student nurses, and where available, clinical officers, medical officers, and the paediatrician on duty. We also collected 10–12 de-identified completed charts from each site to observe them and we used the information gathered as discussion points with the staff. We followed up with the sites through telephone calls to the nurses in charge and data clerks to discuss any issues arising from using the new charts. The proposed changes were discussed within the study team and implemented in the next version of the charts (step 6), repeating in a cyclical manner which resulted in one comprehensive chart- version 2 (Fig. 1). We presented the chart design and piloting progress to the wider CIN-N during two meetings (June 2019 and November 2019) and received feedback from other senior nurses, paediatricians, and health record information officers.

Lastly, at the request of the senior nurses and to support less experienced health workers, we developed a colour-coded information sheet, using input from seven neonatal care experts and published references (47, 48) in March 2020. This information sheet covers normal and out-of-range values for vital signs (temperature, respiratory rate, pulse, oxygen saturation, and blood sugar) and identification of respiratory distress (see Additional file 1: Appendix 1). Hospitals were asked to use the information sheet as a guide and would need to define which actions their health professionals should take if out-of-range values are encountered. The experts also provided feedback which was incorporated into version 2 of the chart.

## Results

### Phase one – understanding user needs, goals and context and tool design

#### Understanding challenges with old charts and the monitoring process (personas and story mapping)

The study team examined filled sample charts from the hospitals and engaged workshop participants using methods from the HCD approach such as personas and an adapted form of user story mapping, to understand challenges with monitoring and chart design.

The primary persona developed was nurse Fiona, often alone or with one or two other colleagues in the ward, caring for up to 50 babies. Clinical Officer John and Medical Officer Intern Thomas were also identified as personas writing the feed and fluid prescriptions as well as reviewing information documented by nurses on the monitoring charts. Both personas undertook many tasks, such as patient care, attending meetings, writing reports, and documenting care. Their common goal was to provide best-practice care to all babies admitted in the newborn units within the time available.

The challenges are generally related to chart design and supply, but also to how charts were used or handled, limited staffing, a knowledge deficit particularly among junior staff, poor interprofessional communication within teams/shifts, and lack of appropriate and working equipment(for example a working pulse oximeter or correct size of the cannula). Some workshop participants reported that mothers were involved in feeding babies and filling feed monitoring charts.

Our observations of filled charts revealed various challenges: i) differences in combination and order of fields across hospitals, ii) different ways of filling the same field by individuals across forms and hospitals, iii) limited space to write values, iii) inadequate fields on forms leading to improvisation, and iv) multiple forms to fill the related information. Additionally, workshop participants through personas and user story mapping identified challenges such as i) multiple forms for the same or related information, ii) a mismatch between equipment calibration and charts, iii) difficult to fill graphs or plain charts, iv) poor inter-professional communication, v) knowledge deficits among junior staff and vi) a mismatch between the order of fields and how tasks are executed. The nurses reported that the order of items in the charts did not follow the order of the tasks they implemented. For example, they may take vital sign measurements before giving feeds or fluids while most charts listed feed/fluids before vital signs. They also felt that for proper feed and fluid monitoring, prescription information should be on the same chart as monitoring information, while prescriptions were often in doctor’s notes or treatment sheets (workshop 1). Doctors echoed the nurses’ suggestions to have everything on one chart but worried it might be too detailed (workshop 2) and said they would consider adopting such a chart if well designed. Interestingly, one nurse claimed that their monitoring chart was perfect. Doctors mentioned that the treatment chart was not adequately designed to document feed or fluid prescriptions but was optimized for drug treatment. Tables 3 and 4 present these challenges.

**Table 3:** Challenges with the monitoring task and charts

Challenge	Description and examples
**Challenges relating to chart design**	
Multiple charts	Participants reported that several charts exist in the wards that collect similar information leading to duplication of information and possibly transcription errors. Current charts that collect feed/fluid prescription and observation data include: 1. Fluid chart 2. Feed chart 3. Weight chart 4. Temperature chart – Respiratory rate, Oxygen saturation, pulse 5. Treatment sheet - feed/fluid prescription 6. Doctors notes – feed/fluid prescription 7. Nursing cardex – may have vitals as well as other notes * In some hospitals, the feed chart also has vital signs
Different ways of filling the same fields	Different ways of filling the same fields make it difficult to interpret what has been recorded and compare between health workers or hospitals; for example, where one uses a plus sign to indicate urine output, could it be interpreted to mean *more* urine was present? Or, where a tick is used, does that imply the presence of urine in the baby’s diaper or that urine output was assessed?
Improvisation due to missing fields	Inadequate fields on forms leading to improvisation; for example, users adding additional columns on the chart or writing over space designated for other fields.
Difficult to fill charts	Limited space to write values – small boxes for filling pulse on temperature charts Current temperature graphs are difficult to fill
A mismatch between units on charts and available equipment	Example: temperature scale in Fahrenheit while thermometers show the temperature in centigrade
Different charts to write related information	Participants felt that for proper feed and fluid monitoring, prescription information should be on the same chart as monitoring information as opposed to having the prescription in the doctor’s notes or the treatment sheet.
Charts do not support workflow	The charts were not designed to support the order nurses follow while carrying out their tasks. For example, they typically bundle tasks; take vital signs measurements before giving feeds or fluids while some charts listed the feed/fluids before the vital signs.
**General monitoring challenges**	
Interprofessional communication	Poor interprofessional communication was reported as a challenge to monitoring by both nurses and doctors as well as between junior and senior professionals within the cadres.
Knowledge deficit	Both doctors and nurses felt that less experienced health workers such as interns lacked knowledge in prescribing (particularly for low birth weight babies) and identification of problems (for example out of range values) possibly due to their limited experience.
Staff shortage	Nurses felt that staff shortage was a barrier to the proper documentation. Related to this, some hospitals reported that mothers were involved in feeding babies and filling feed monitoring charts
Charts shortage	The participants reported an inadequate supply of charts
Misplaced charts	Nurses reported that in many cases, monitoring charts would either be placed at the bedside or at the nursing station in a pile which sometimes led to charts being misplaced.

**Table 4:**
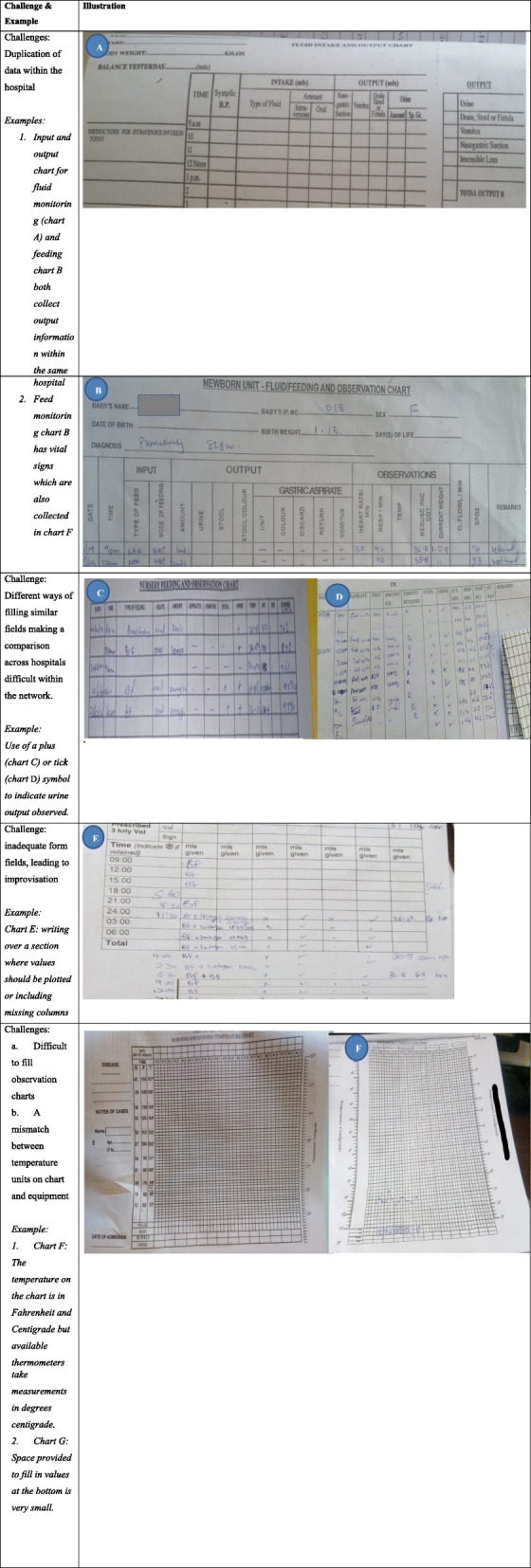
Challenges with current vital signs, feeds and fluids monitoring forms used in newborn units within CIN-N

The adapted user story mapping in workshops 2 and 3 revealed that the monitoring process occurred in two stages. First, the feed and/or fluid prescription task involved calculating the prescription and writing it in the doctor’s notes and/or treatment sheet. Second, the patient monitoring task based on the baby’s condition generally followed five steps: identification of the correct patient, clinical assessment, administering feeds and/or fluids, writing findings and actions on the chart, and reviewing documentation.

From workshops 2 and 3, it emerged that the feed/fluid prescription was written multiple times by the doctor (in the doctor’s notes as well as the treatment sheet) and then transcribed to the monitoring sheet by the nurse. The nurse translates the doctor’s prescription into an actionable plan. For example, the doctor writes a fluid prescription for 24 h, but the nurses need to calculate how much is required 6-hourly or 8-hourly based on equipment available or work plan; some paediatricians likened this dilemma to a broken telephone where information gets distorted each time it is passed on to the next person. The user story mapping in workshop 3 uncovered the challenges encountered during monitoring. Nurses noted that problems (for example out of range values) are recognized through the review of separate charts (Table 3), but the recommended action might be documented in the nursing notes; they considered this time-consuming and overwhelming. Additionally, both clinicians and nurses (workshops 2 and 3) felt that less experienced health workers such as interns lacked knowledge in prescribing and identifying problems (for example out of range values), likely due to limited experience. This was aggravated by the lack of proper communication between cadres and fear of reporting problems, identified in all workshops.

##### Designing together

The nurses and paediatricians wanted one chart to capture vital signs, assessment of the baby and feed and fluid prescription and monitoring. They suggested that the chart should have a biodata section above and a notes section below. Nurses in workshop 3 also felt that all staff needed to take responsibility or ownership of their care by signing the documentation after each bundle of tasks; apparently, nurses sometimes organized care by splitting tasks, whereby vital signs and assessment were done by one person, and feed and fluid monitoring by another. The decision to have each nurse sign for their bundle of tasks as opposed to one nurse signing for all tasks and thereby assuming responsibility was preceded by a discussion on adopting primary care nursing as a model. In this model, a single nurse is identified as the point of contact during the entire patient stay; that nurse accepts responsibility for all care provided [49]. However, this model is not widely implemented in the hospitals represented, so the nurses agreed that each nurse should sign for his/her tasks. Both nurses and doctors suggested that the chart should span several days, especially as printing and paper supplies are limited. Lastly, to reduce the time spent on writing, the chart should include fixed options. The charts were designed to fit A4 size paper, landscape orientation, and printed on both sides. Implementing these new charts would imply that hospitals could reduce the number of pieces of paper where related information is written from seven to one. For example, the feed and fluid prescriptions were previously written in the doctor’s notes, treatment sheet, and feed monitoring chart, but can now be written in the prescription section of the monitoring chart.

##### Monitoring charts version 1

The study team designed two charts to cater to the needs of babies requiring varying monitoring frequency. The standard monitoring chart could be used over 4 days while the intensive monitoring chart covered 2 days with hourly slots. Table 5 highlights the differences between the two charts and a sample of the charts is provided in the appendices [see Additional file 2: Appendix 2.1 and 2.2].

**Table 5:** Standard and intensive monitoring chart differences

	Time	Frequency of monitoring	Duration of one sheet of paper
Standard monitoring chart	Timings fixed 3 hrly for 24 h period 9 am, 12Midday, 3 pm, 6 pm, 9 pm, 12Midnight, 3 am and 6 am	3hrly monitoring to less frequent monitoring	Spans 4 days
Intensive monitoring chart	Timings fixed hourly from 7 AM to 6 AM for 24 h period	Flexible to monitor babies hourly if needed.	Spans 2 days

### Phase two -piloting and changing

**Fig. 2:**
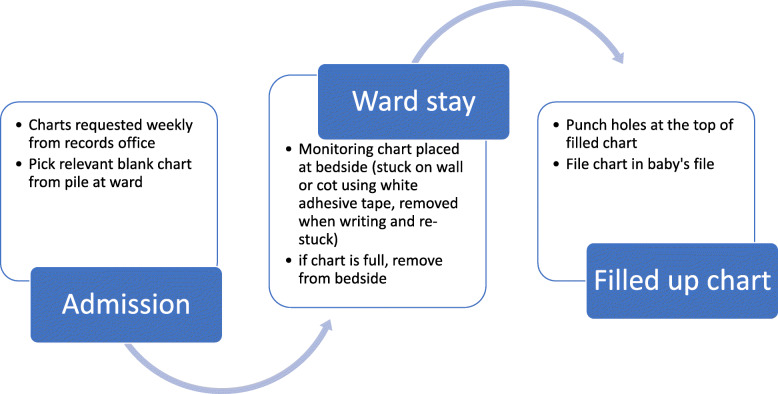
General process flow of charts

From the workshop discussions and piloting sessions, we sought to understand how charts are moved from one location to another in the ward and potential loss, a challenge identified in workshop 3. In one pilot hospital, monitoring charts were kept at the nursing station in a pile before use, placed by the bedside while in use, and then inserted into the patient’s file when filled. In another county hospital, bound booklets with all charts required for each patient in the newborn unit were provided. Additional charts were attached to the back of the bound booklets using white elastic adhesive tape Fig. 2.

#### Feedback on version 1 charts

Overall, the staff at the hospital (nurses, paediatricians, medical officers, clinical officers, interns, and students present on the day of the visit) who had used the chart were happy that it covered all they needed to monitor on one page. Medical officers and paediatricians especially noted that they previously had challenges learning what happened to the baby and the volume of feeds given, now the chart allowed them to see how the baby was progressing without having to look through several documents in the patient’s file. The hospital staff gave feedback on chart layout and suggested design improvements such as re-ordering fields, adding missing fields, and increasing space for feed and fluid prescription. There were concerns about the flexibility of the standard monitoring chart given the fixed timings at the top. For feed monitoring, a paediatrician suggested asking mothers to document the feeds they gave on the same chart. Nurses were concerned about how the mothers would perceive being asked to document feeds but were willing to support them if the request came from a senior authority, for example, the paediatrician. They suggested that mothers could use a simple chart that could be inserted into the patient’s file.

During the piloting sessions, we obtained de-identified copies of filled charts and observed challenges with chart handling and staffing. In one hospital, before the chart was filled up, it was stuck on the baby’s cot using white elastic adhesive tape, then removed and filed into the baby’s file after holes were punched at the top. Important information at the top of the chart was lost when taping the charts on and off or when holes were punched at the top. We observed that with subsequent photocopies of photocopied charts, information on the margins was cut off. Regarding filling the charts, one hospital managed to perform 6-hourly monitoring, citing staff shortages, while another had charts that were filled at 3-hourly intervals. During the piloting period, they had nursing students but explained that otherwise, they could not have achieved the same level of documentation (during that site visit, 40 babies were admitted, and three qualified nurses were on duty for 24 h).

##### Monitoring chart version 2

During the pilot visits, nurses and paediatricians emphasized the need for a proper feed and fluid prescription section and suggested that a larger prescription section be added. A senior nurse suggested reducing the number of days covered by the chart and instead incorporating the prescription. The version 1 chart had a small section where the nurse would transfer the prescription; this did not overcome the earlier-identified challenge of duplication. Additionally, it emerged that printing two charts and ensuring that each baby has the correct chart would be a logistical challenge. The standard monitoring chart did not offer flexibility in case a baby’s condition deteriorated or if the baby’s review differed from the fixed schedule indicated. Therefore, we merged the two charts to develop a Comprehensive Newborn Monitoring Chart with no fixed timings at the top to allow flexibility. The version 2 chart (see Additional file 3: Appendix 3) is a 2-day chart with the prescription on the left-hand side and monitoring fields on the right that can be used over 48 h if printed in duplex. The monitoring section on the right now had a larger space to write observations.

##### Reflection on the design process

We set out to design a monitoring chart that we anticipated would be used by nurses to document the care they provide to babies admitted in the newborn unit, as they have the most frequent contact with the babies. We aimed to put the needs of the users first and therefore adopted the HCD approach, to understand their needs and context, co-design, and pilot the charts. It emerged that the nurses preferred to have all information related to the monitoring task on one page, leading to a comprehensive newborn monitoring chart to be used by nurses and doctors. The approach allowed the design team and stakeholders to focus on the user to develop a context-appropriate chart and identify any problems early during the design and pilot phases. The workshop participants demonstrated enthusiasm and confidence, suggesting design features and supporting them with illustrations from their workplaces. During piloting, nurses were eager to use the chart, as demonstrated by the volunteers who opted to test the chart in their facility even before the design team selected a pilot site. They were also proactive in printing additional copies of charts and gathering their staff for feedback sessions even with their busy schedules.

During the workshops, the study team encouraged participants to consider the practicality of their suggestions, bearing in mind that they were likely to have the most contact with patients. The participants also had to consider the amount of space available on the A4 sheet; more items implied less space for other items or smaller space for values. The study team and workshop participants converged on practical proposals even as aspirational proposals were made (step 2–5) throughout the design and piloting sessions. The most documented items were included, leaving space for more detailed assessments or examinations in the notes section or nursing Cardex, for a comprehensive picture of patients’ progress. This design would also support less experienced staff to remember what must be documented for all babies.

Throughout the discussions, we aimed to build consensus among participants’ differing opinions by referring to clinical guidelines, rather than what has been the norm at the hospitals, to avoid propagating poor practice. For example, some hospitals were routinely doing gastric aspirates for all babies, while others were not; the version 1 chart included a row to document if aspirates were done. However, upon review of evidence by a paediatrics training team, it emerged that regular aspirates were not beneficial [50–53]. With this evidence, the nurse managers agreed to remove that row from the chart. In a recent follow-up meeting, the nurses agreed that best practice must be encouraged through the charts, in their hospitals. In our case, it was important to design for context, but also with clinical evidence in mind.

## Discussion

Charts used in documenting newborn nursing care have received little attention, resulting in the widespread use of poorly designed monitoring charts. To overcome this, we adapted and used a HCD approach to uncover challenges with existing charts and the monitoring process, co-design and pilot a new context-appropriate chart with staff from hospitals participating in a clinical information network. We found a variety of charts in use within and across hospitals and identified challenges related to chart content or design features, chart handling, and limited resources. These design challenges may hamper hospitals’ efforts to provide high-quality care to admitted newborns which is a critical element in reducing neonatal mortality.

The workshops revealed that health workers were required to write related information in multiple locations, transfer feed and fluid prescriptions up to three times, and write information multiple times in different charts. Transferring information can introduce transcription errors which in turn may affect the care provided. Additionally, duplication of efforts contributes to workflow inefficiencies in an already resource-constrained environment. It was suggested that one monitoring chart should capture multiple monitoring activities (vital signs, feed, and fluid prescription, and monitoring) likely to be done for most babies, to be used over several days. This design was highly appreciated by the physicians, as they could ‘see’ the baby’s progress in one location without having to search the patient’s file for important information. They were concerned about the level of detail in a comprehensive chart. In contrast, observation charts found in the literature, used during the inpatient stay, do not typically combine various monitoring tasks but only focus on vital signs [23, 54–56]. This makes our chart design unique.

If the newly designed charts are adopted, they will reduce the number of places where related information is recorded from as many as seven to one. Additionally, when the chart is printed on both sides of an A4 sheet, one can be used for one baby over 48 h (2-6hourly), which helps to reduce the number of charts to be printed and reduces demand on limited resources. The study team had anticipated re-designing several charts (feeding chart, fluid chart, and observation chart for vital signs) but workshop participants emphasized that they would prefer if all related information was in one place. This chart design can be adapted by other hospitals in low- and middle-income settings but might not be appropriate for high-income settings with more resources available to print colour charts [57] or use electronic systems [58, 59].

Well-designed paper-based medical records that are acceptable to staff may improve communication and foster teamwork, generating information, and sharing for good monitoring and quality of care. They can prepare the ground for future electronic medical records [60]. Charts contribute to improved documentation efficiency thereby increasing time for key clinical activities. Harmonization of forms can ease the orientation of new staff across different facilities and may promote quality gains and reduce chances of errors [61]. Current charts do not facilitate staff movement between hospitals, as changing hospitals often means learning to use different charts. Adopting structured monitoring charts within the CIN-N facilitates the generation of clear information on how care is provided for newborns across the network and provides opportunities to identify areas of quality improvement backed by data, which is a CIN-N goal. We hope to facilitate building a clearer picture of newborn care and the provision of best-practice care, by making nursing care ‘visible’.

The involvement of mothers in inpatient newborn care has been highlighted in a recent study that explored the nature of task-sharing [62]. However, their involvement in documentation, such as, recording feeds, remains largely unexplored. Nurses recommended that the mothers be given a simple chart to record their feeding; a pilot hospital received this suggestion with caution. Patient records are legal documents and the implications need clarification. Considering that task-sharing is gaining ground as a possible solution to staff shortages, documentation of care by mothers could be explored to understand the perceptions of both health workers and mothers.

Designing a new chart does not overcome the process issues, health worker shortages, lack of knowledge, and skill deficiencies identified as challenges to good monitoring. Process challenges such as how the charts are handled in the ward and how supplies (appropriate equipment and charts) are sourced and maintained will require different solutions. They might involve an examination of how care is organized and how this affects the care provided; ward managers could use this information to advocate for more resources and improve their processes. Knowledge or skill deficiencies can be addressed through targeted continuous medical education as information becomes available through filled charts and documentation audits. There is an opportunity to intervene during the chart implementation period and to try to address issues arising as we conduct an evaluation guided by theory. One such theory is the Normalization Process Theory, which provides a framework for thinking about implementation, sustainability, and evaluation of interventions using four main components (sense-making, engagement, collective action, and reflexive monitoring) to articulate how interventions become assimilated into practice [63]. It can suggest where bottlenecks might occur, to address the issues from the start rather than transferring existing routines. For example, during problem identification, the nurses reported that charts may be kept at the bedside during inpatient stays; piloting at one hospital confirmed that. However, observations and discussions at pilot sites showed that white adhesive was used to stick the chart to the baby’s cot, covering important information at the top and leading to tears upon its removal. Identifying solutions to such challenges may ease the process of implementing the new charts.

Limited evidence is emerging from high-income countries such as Australia that focus on designing observation and response charts [64, 65] that incorporate a human-factors approach. We believe that the personas and user-stories embedded in the HCD approach we adopted to develop paper-based charts was novel in our setting. In designing the charts together with the users and involving more stakeholders, we anticipate that the process will contribute to building a sense of ownership and increasing the uptake of new structured charts to better document care. We will conduct a mixed-methods evaluation to understand the uptake of charts at hospitals as well as the perceptions of health workers on the new charts.

### Limitations

Nurses were only available together at one place for limited periods; given their already high workloads, it was impossible to hold longer workshops for extensive exploration and development of chart design. The limited time ensured a strong focus on obtaining a prototype that could quickly be tested in hospitals.

## Conclusions

We used a Human-Centred approach to design a unique inpatient newborn monitoring chart for a network of hospitals. The current monitoring charts had problems related to content and a poor design hampering documentation of the care provided. Other problems were identified, related to process - how care was organized in the newborn ward, lack of resources (appropriate equipment, staff, and charts), knowledge deficits among less experienced staff, and poor interprofessional communication. The newly designed chart is a comprehensive monitoring chart covering feed and fluid prescription, input, and output monitoring, and vital sign monitoring, which is quite different from observation charts in the literature that focus only on vital signs. While the chart designed does not address all the issues raised, we believe that once implemented, it will facilitate building a clearer picture of the care given to newborns and in turn, facilitate the provision of best-practice care by making the nursing care provided ‘visible’. We will conduct a mixed-methods evaluation to assess the uptake of charts and documentation outcomes build a comprehensive picture of the entire process and identify potential points for intervention.

## Supplementary Information

**Additional file 1: Appendix 1.**. Information sheet.

**Additional file 2: Appendix 2.1.** Neonatal standard monitoring charts version 1. Appendix 2.2 Neonatal intensive monitoring charts version 1.

**Additional file 3: Appendix 3.** Comprehensive Newborn Monitoring Chart.

## Data Availability

Data sharing is not applicable to this article as no datasets were generated or analysed during the current study.

## References

[1] David Sharrow LH, Liu Y, You D, on behalf of the United Nations Inter-agency Group for Child Mortality Estimation (UN IGME) (2020). Levels and trends in child mortality 2020. UNICEF headquarters: United Nations Inter-agency Group for Child Mortality Estimation (UN IGME).

[2] United Nations (2015). Goal 3: ensure healthy lives and promote well-being for all at all ages: United Nations.

[3] WHO (2019). Children: reducing mortality: WHO.

[4] Doctor HV, Radovich E, Benova L (2019). Time trends in facility-based and private-sector childbirth care: analysis of Demographic and Health Surveys from 25 sub-Saharan African countries from 2000 to 2016. J Glob Health.

[5] Lawn JE, Kinney MV, Black RE, Pitt C, Cousens S, Kerber K (2012). Newborn survival: a multi-country analysis of a decade of change. Health Policy Plan.

[6] Barbosa VM (2013). Teamwork in the neonatal intensive care unit. Phys Occup Ther Pediatr.

[7] World Health Organization (2019). Survive and thrive: transforming care for every small and sick newborn.

[8] Murphy GAV, Omondi GB, Gathara D, Abuya N, Mwachiro J, Kuria R, Tallam-Kimaiyo E, English M (2018). Expectations for nursing care in newborn units in Kenya: moving from implicit to explicit standards. BMJ Glob Health.

[9] Ricci SS (2013). Essentials of maternity, newborn, & Women’s health nursing: Wolters Kluwer health | Lippincott Williams & Wilkins.

[10] Moxon SG, Lawn JE, Dickson KE, Simen-Kapeu A, Gupta G, Deorari A, Singhal N, New K, Kenner C, Bhutani V, Kumar R, Molyneux E, Blencowe H (2015). Inpatient care of small and sick newborns: a multi-country analysis of health system bottlenecks and potential solutions. BMC Pregnancy Childbirth.

[11] Knebel E, Lundahl S, Edward A, Raj HA, Ashton J, Wilson N. The Use of Manual Job Aids by Health Care Providers: What Do We Know?2000; (1). Available from: https://www.usaidassist.org/sites/assist/files/use_of_job_aids_qap_2000.pdf.

[12] Hallam BD, Kuza CC, Rak K, Fleck JC, Heuston MM, Saha D, Kahn JM (2018). Perceptions of rounding checklists in the intensive care unit: a qualitative study. BMJ Qual Saf.

[13] Mwakyusa S, Wamae A, Wasunna A, Were F, Esamai F, Ogutu B (2006). Implementation of a structured paediatric admission record for district hospitals in Kenya--results of a pilot study. BMC Int Health Hum Rights.

[14] WHO (2015). WHO Safe Childbirth Checklist.

[15] Semrau KEA, Hirschhorn LR, Marx Delaney M, Singh VP, Saurastri R, Sharma N, Tuller DE, Firestone R, Lipsitz S, Dhingra-Kumar N, Kodkany BS, Kumar V, Gawande AA, BetterBirth Trial Group (2017). Outcomes of a coaching-based WHO safe childbirth checklist program in India. N Engl J Med.

[16] Abraham J, Kannampallil T, Patel VL (2014). A systematic review of the literature on the evaluation of handoff tools: implications for research and practice. J Am Med Inform Assoc.

[17] Wagoro MCA, Rakuom CP (2015). Mainstreaming Kenya-nursing process in clinical settings: the case of Kenya. Int J Africa Nurs Sci.

[18] Cowden S, Johnson LC (2004). A process for consolidation of redundant documentation forms. Comput Inform Nurs.

[19] Chelagat D, Sum T, Obel M, Chebor A, Kiptoo R (2013). PriscahBundotich-Mosol. Documentation: Historical Perspectives, Purposes, Benefits and Challenges as Faced by Nurses. Int J Humanit Soc Sci.

[20] Hardey M, Payne S, Coleman P (2000). ‘Scraps’: hidden nursing information and its influence on the delivery of care. J Adv Nurs.

[21] Hendrich A, Chow MP, Skierczynski BA, Lu Z (2008). A 36-hospital time and motion study: how do medical-surgical nurses spend their time?. Perm J.

[22] Yen P-Y, Kellye M, Lopetegui M, Saha A, Loversidge J, Chipps EM, Gallagher-Ford L, Buck J (2018). Nurses' time allocation and multitasking of nursing activities: a time motion study. AMIA Annu Symp proc.

[23] Chatterjee MT, Moon JC, Murphy R, McCrea D (2005). The “OBS” chart: an evidence based approach to re-design of the patient observation chart in a district general hospital setting. Postgrad Med J.

[24] McKay H, Mitchell IA, Sinn K, Mugridge H, Lafferty T, Van Leuvan C (2013). Effect of a multifaceted intervention on documentation of vital signs and staff communication regarding deteriorating paediatric patients. J Paediatr Child Health.

[25] Ogero M, Ayieko P, Makone B, Julius T, Malla L, Oliwa J (2018). An observational study of monitoring of vital signs in children admitted to Kenyan hospitals: an insight into the quality of nursing care?. J Glob Health.

[26] Preece M, Horswill M, Hill A, Karamatic R, Hewett D, Watson M. Heuristic Analysis of 25 Adult General Observation Charts. St Lucia: Australian Commission on Safety and Quality in Health Care; 2009.

[27] Aluvaala J, Nyamai R, Were F, Wasunna A, Kosgei R, Karumbi J, Gathara D, English M, SIRCLE/Ministry of Health Hospital Survey Group (2015). Assessment of neonatal care in clinical training facilities in Kenya. Arch Dis Child.

[28] Bazzano AN, Martin J, Hicks E, Faughnan M, Murphy L (2017). Human-centred design in global health: a scoping review of applications and contexts. PLoS One.

[29] Norman DA (2013). The Design of Everyday Things: revised and expanded edition: basic books.

[30] IDEO.org. The Field Guide to Human-Centered Design. Canada: IDEO.org; 2015.

[31] Brown T (2010). Design thinking for social innovation. Development Outreach.

[32] Kim S, Piccinini D, Mensah E, Lynch M (2019). Using a human-centered design approach to determine consumer preferences for long-lasting insecticidal nets in Ghana. Glob Health Sci Pract.

[33] Seffah A, Gulliksen J, Desmarais MC (2005). Human-centered software engineering — integrating usability in the software development lifecycle.

[34] Macdonald M, Putzer T (2019). Human-centered design and sustainable malaria interventions. Glob Health Sci Pract.

[35] Boyd H, McKernon S, Mullin B, Old A (2012). Improving healthcare through the use of co-design. J N Z Med Assoc.

[36] Bowen S, Sustar H, Wolstenholme D, Dearden A (2013). Engaging teenagers productively in service design. Int J Child-Comput Interact.

[37] Beck K, Beedle M, Av B, Cockburn A, Cunningham W, Fowler M (2001). Principles behind the Agile Manifesto.

[38] Gothelf J, Seiden J (2013). Lean UX: Applying Lean Principles to Improve User Experience O'Reilly Media.

[39] Gibbons S (2018). Journey mapping 101: Nielsen Norman Group.

[40] Interaction Design Foundation (2020). User Stories: Interaction Design Foundation.

[41] Tuti T, Bitok M, Malla L, Paton C, Muinga N, Gathara D, et al. Improving documentation of clinical care within a clinical information network: an essential initial step in efforts to understand and improve care in Kenyan hospitals. BMJ Global Health. 2016;1(1):e000028. 10.1136/bmjgh-2016-000028 PMC4934599 27398232

[42] Tuti T, Bitok M, Paton C, Makone B, Malla L, Muinga N, Gathara D, English M (2015). Innovating to enhance clinical data management using non-commercial and open source solutions across a multi-center network supporting inpatient pediatric care and research in Kenya. J Am Med Inform Assoc.

[43] Ayieko P, Ogero M, Makone B, Julius T, Mbevi G, Nyachiro W, Nyamai R, Were F, Githanga D, Irimu G, English M, Clinical Information Network authors (2016). Characteristics of admissions and variations in the use of basic investigations, treatments and outcomes in Kenyan hospitals within a new clinical information network. Arch Dis Child.

[44] Gathara D, Serem G, Murphy GAV, Obengo A, Tallam E, Jackson D, Brownie S, English M (2020). Missed nursing care in newborn units: a cross-sectional direct observational study. BMJ Qual Amp Saf.

[45] Dam RF, Teo YS (2020). Personas – a simple introduction interaction Design Foundation.

[46] Ogero M, Akech S, Malla L, Agweyu A, Irimu G, English M (2020). Examining which clinicians provide admission hospital care in a high mortality setting and their adherence to guidelines: an observational study in 13 hospitals. Arch Dis Child.

[47] Mulvihill M, EOE Neonatal ODN NATT Tool Working Group (2018). Clinical Guideline: Neonatal Alert, Trigger & Track (NATT) tool.

[48] World Health Organisation (2013). Thermal Control of the Newborn: a practical guide.

[49] Tiedeman M, PhD RN, Lookinland S, PhD RN (2004). Traditional models of care delivery: what have we learned?. J Nurs Adm.

[50] Abiramalatha T, Thanigainathan S. B N Routine monitoring of stomach aspirates for prevention of necrotising enterocolitis in preterm infants. Cochrane Database of Syst Rev. 2019;7. 10.1002/14651858.CD012937.pub2. 10.1002/14651858.CD012937.pub2 PMC6699661 31425604

[51] Dutta S, Singh B, Chessell L, Wilson J, Janes M, McDonald K, Shahid S, Gardner V, Hjartarson A, Purcha M, Watson J, de Boer C, Gaal B, Fusch C (2015). Guidelines for feeding very low birth weight infants. Nutrients.

[52] Kaur A, Kler N, Saluja S, Modi M, Soni A, Thakur A, Garg P (2015). Abdominal circumference or gastric residual volume as measure of feed intolerance in VLBW infants. J Pediatr Gastroenterol Nutr.

[53] Li YF, Lin HC, Torrazza RM, Parker L, Talaga E, Neu J (2014). Gastric residual evaluation in preterm neonates: a useful monitoring technique or a hindrance?. Pediatr Neonatol.

[54] Cahill H, Jones A, Herkes R, Cook K, Stirling A, Halbert T, Yates A, Lal S, Gardo A, Donnelly R, Gattas DJ, on behalf of the Royal Prince Alfred Hospital Clinical Emergency Response System Steering Committee (2011). Introduction of a new observation chart and education programme is associated with higher rates of vital-sign ascertainment in hospital wards. BMJ Qual Saf.

[55] Robb G, Seddon M (2010). A multi-faceted approach to the physiologically unstable patient. Qual Saf Health Care.

[56] Muinga N, Abejirinde I-OO, Paton C, English M, Zweekhorst M. Designing paper-based records to improve the quality of nursing documentation in hospitals: A scoping review. J Clin Nurs. 10.1111/jocn.15545 PMC7894495 33113237

[57] Horswill MS, Preece MHW, Hill A, Christofidis MJ, Karamatic R, Hewett D, et al. Human factors research regarding observation charts: research project overview: School of Psychology. St Lucia: The University of Queenslandv; 2010.

[58] Atasoy H, Greenwood BN, McCullough JS (2019). The digitization of patient care: a review of the effects of electronic health records on health care quality and utilization. Annu Rev Public Health.

[59] Bonomi S (2016). The Electronic Health Record: A Comparison of Some European Countries. Information and Communication Technologies in Organizations and Society.

[60] Mann R, Williams J (2003). Standards in medical record keeping. Clin Med.

[61] Christofidis MJ, Hill A, Horswill MS, Watson MO (2013). A human factors approach to observation chart design can trump health professionals' prior chart experience. Resuscitation.

[62] Omondi GB, Murphy GAV, Jackson D, Brownie S, English M, Gathara D (2020). Informal task-sharing practices in inpatient newborn settings in a low-income setting—a task analysis approach. Nurs Open.

[63] Murray E, Treweek S, Pope C, MacFarlane A, Ballini L, Dowrick C, Finch T, Kennedy A, Mair F, O'Donnell C, Ong BN, Rapley T, Rogers A, May C (2010). Normalisation process theory: a framework for developing, evaluating and implementing complex interventions. BMC Med.

[64] Preece MHW, Hill A, Horswill MS, Dunbar N, Adams LM, Stephens J-AL (2010). Developer’s guide for observation and response charts.

[65] Australian Commission on Safety and Quality in Health Care (2019). Observation and response charts: Australian Commission on Safety and Quality in Health Care.

