# Supplementary figures and images for: Using a human-centred design approach to develop a comprehensive newborn monitoring chart for inpatient care in Kenya
Source: BMC Health Serv Res. 2021 Sep 24;21:1010. doi: 10.1186/s12913-021-07030-x (PMC8461871; doi:10.1186/s12913-021-07030-x)

## Appendix 1 Information sheet


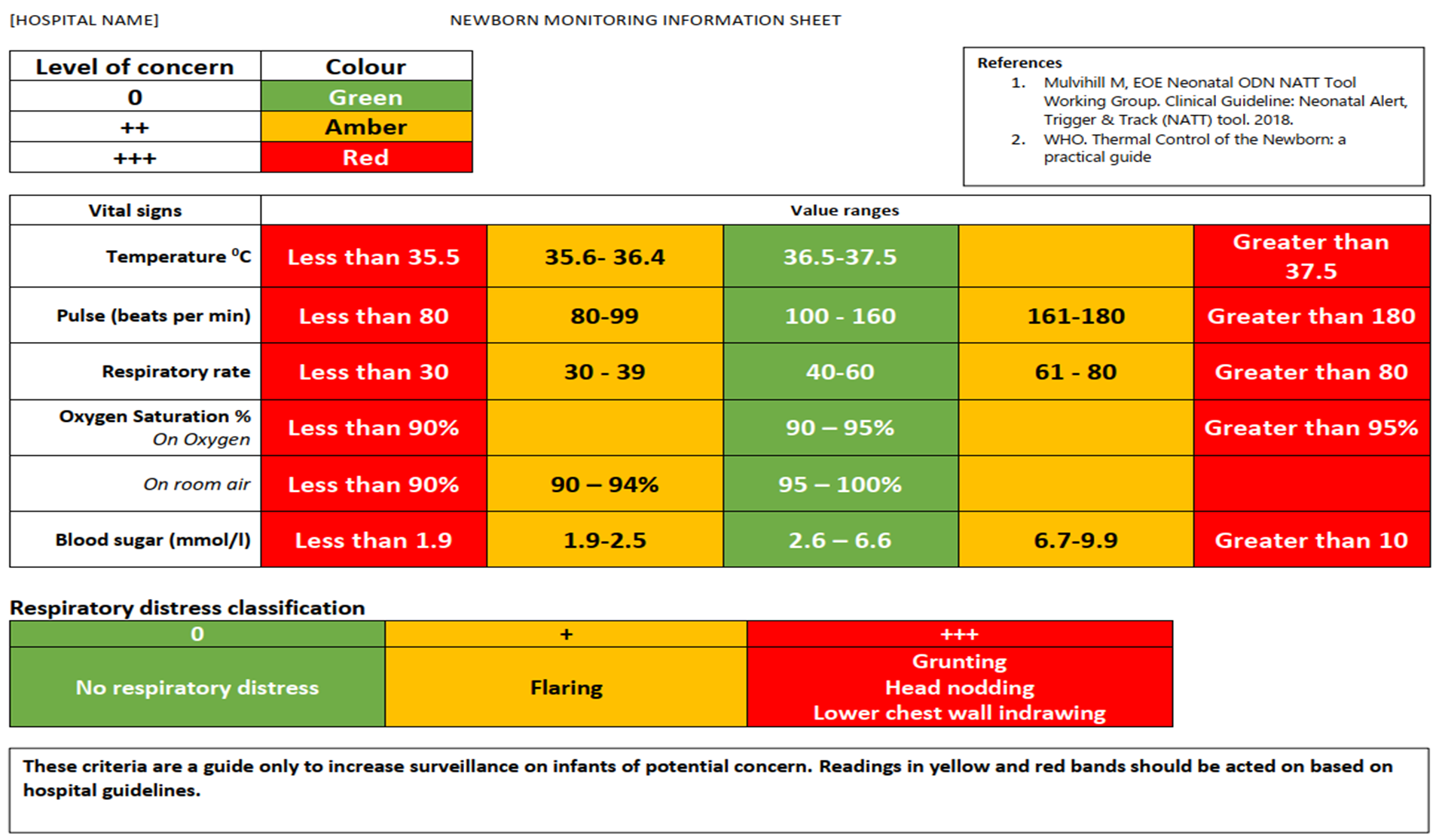

Supplement: Supplementary file 1 — Additional file 1: Appendix 1.. Information sheet. [file 12913_2021_7030_MOESM1_ESM.docx]

##
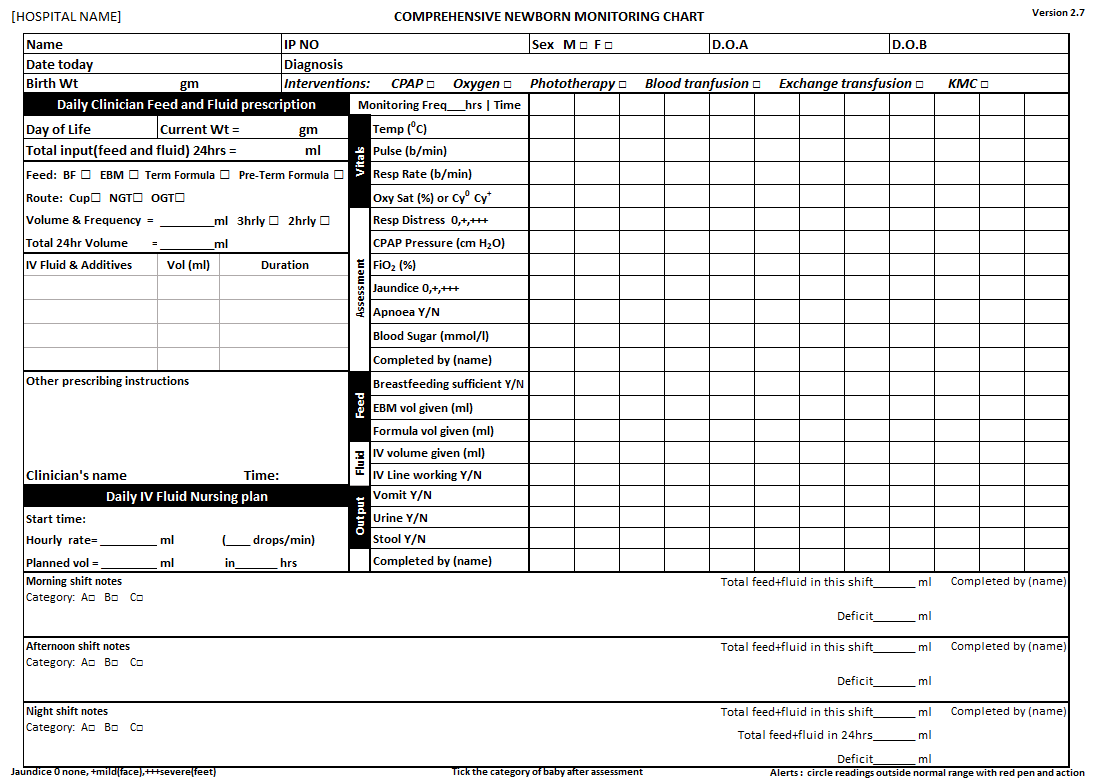
Appendix 3 Comprehensive Newborn Monitoring Chart

Supplement: Supplementary file 3 — Additional file 3: Appendix 3. Comprehensive Newborn Monitoring Chart. [file 12913_2021_7030_MOESM3_ESM.docx]
