# Supplementary material for: Using a human-centred design approach to develop a comprehensive newborn monitoring chart for inpatient care in Kenya
Source: BMC Health Serv Res. 2021 Sep 24;21:1010. doi: 10.1186/s12913-021-07030-x (PMC8461871; doi:10.1186/s12913-021-07030-x)

## Appendix 2.1 Neonatal standard monitoring charts version 1


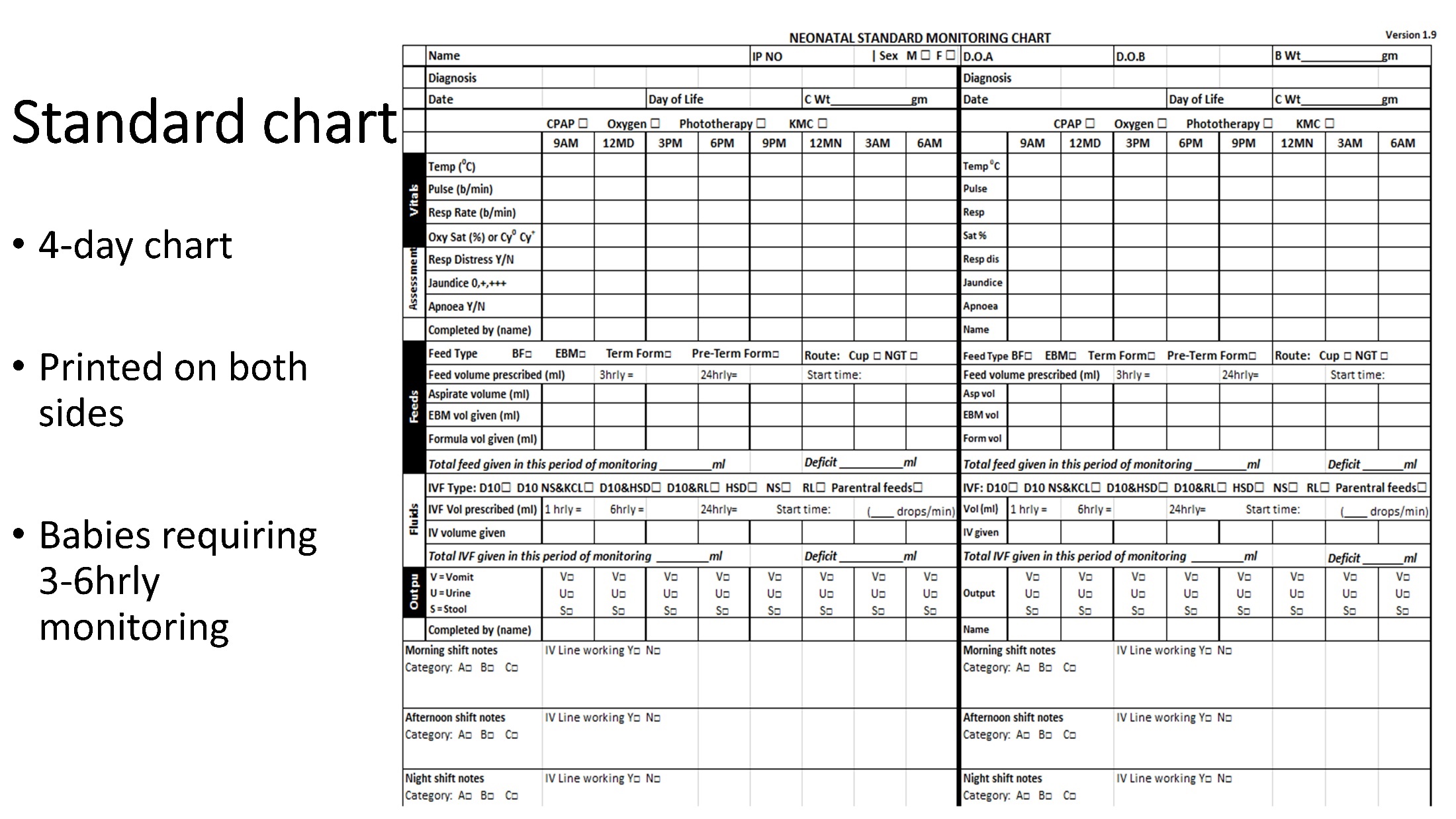


## Appendix 2.2 Neonatal intensive monitoring charts version 1
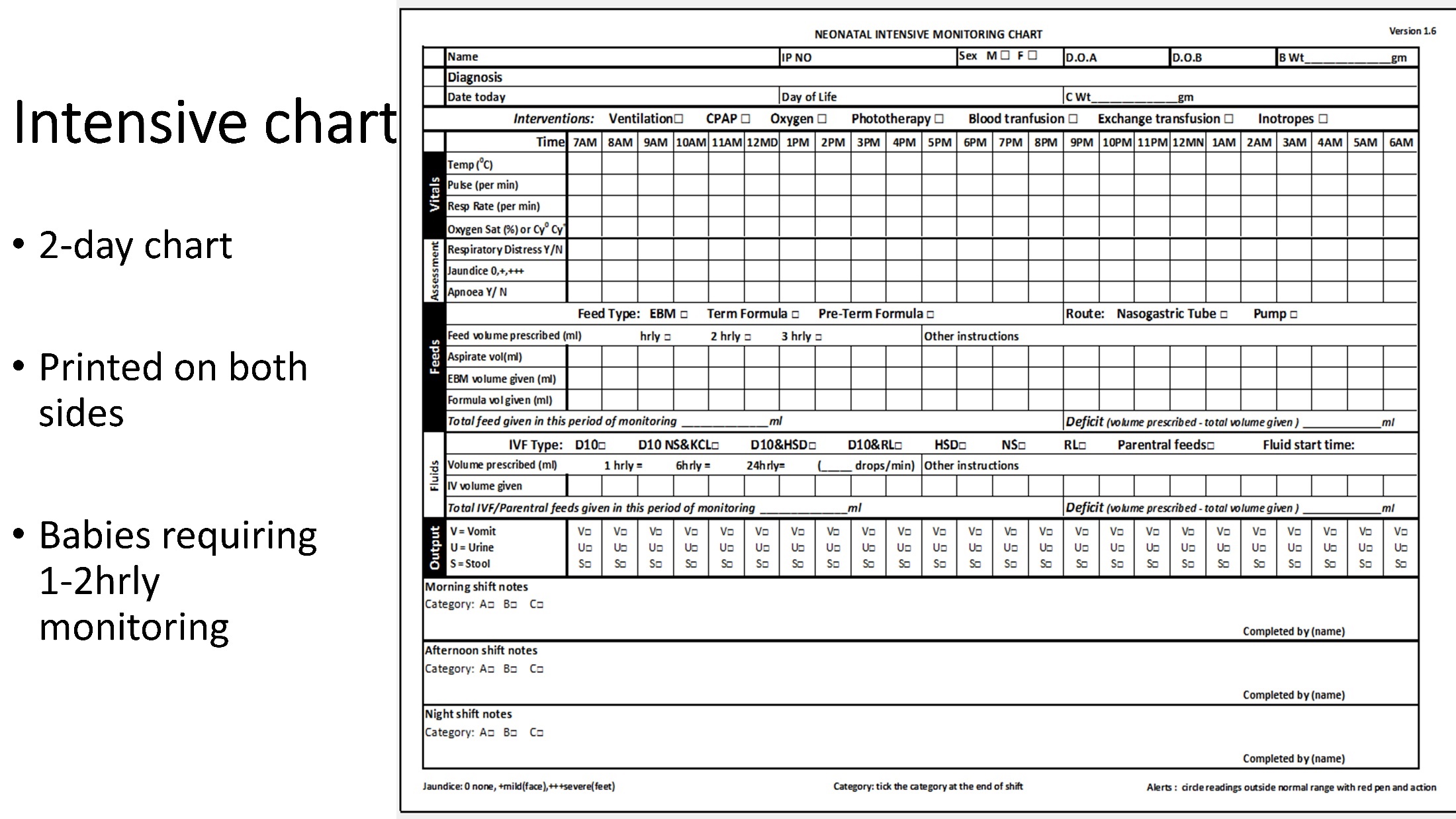

Supplement: Supplementary file 2 — Additional file 2: Appendix 2.1. Neonatal standard monitoring charts version 1. Appendix 2.2 Neonatal intensive monitoring charts version 1. [file 12913_2021_7030_MOESM2_ESM.docx]
